# Genetic characterization and molecular epidemiology of Coxsackievirus A12 from mainland China during 2010–2019

**DOI:** 10.3389/fmicb.2022.988538

**Published:** 2022-12-21

**Authors:** Qin Guo, Hehe Zhao, Yong Zhang, Xianjun Wang, Qiuli Yu, Zhaolin Tan, Huanhuan Lu, Jinbo Xiao, Tianjiao Ji, Shuangli Zhu, Dongyan Wang, Qian Yang, Zhenzhi Han, Wenbo Xu, Dongmei Yan

**Affiliations:** ^1^National Polio Laboratory, WHO WPRO Regional Polio Reference Laboratory, National Health Commission Key Laboratory for Biosecurity, National Health Commission Key Laboratory for Medical Virology, Chinese Center for Disease Control and Prevention Beijing, National Institute for Viral Disease Control and Prevention, Beijing, China; ^2^Da Zhou Vocational College of Chinese Medicine, Dazhou, China; ^3^Center for Biosafety Mega-Science, Chinese Academy of Sciences, Wuhan, China; ^4^Shandong Center for Disease Control and Prevention, Shandong, China; ^5^Hebei Center for Disease Control and Prevention, Shijiazhuang, China; ^6^Tianjin Center for Disease Control and Prevention, Tianjin, China

**Keywords:** Coxsackievirus A12, phylogenetic analysis, evolutionary dynamics, recombination, hand, foot and mouth disease, enteroviruses

## Abstract

Coxsackievirus A12 (CVA12) is an enterovirus that has been isolated in many countries in recent years. However, studies on CVA12 are limited, and its effective population size, evolutionary dynamics and recombination patterns have not been clarified now. In this study, we described the phylogenetic characteristics of 16 CVA12 strains isolated from pediatric HFMD patients in mainland China from 2010 to 2019. Comparison of the nucleotide sequences and amino acid sequences with the CVA12 prototype strain revealed that the 16 CVA12 strains are identical in 78.8–79% and 94–94.2%, respectively. A phylodynamic analysis based on the 16 full-length VP1 sequences from this study and 21 sequences obtained from GenBank revealed a mean substitution rate of 6.61 × 10^−3^ substitutions/site/year (95% HPD: 5.16–8.20 × 10^−3^), dating the time to most recent common ancestor (tMRCA) of CVA12 back to 1946 (95% HPD: 1942–1947). The Bayesian skyline plot showed that the effective population size has experienced twice dynamic fluctuations since 2007. Phylogeographic analysis identified two significant migration pathways, indicating the existence of cross-provincial transmission of CVA12 in mainland China. Recombination analysis revealed two recombination patterns between 16 CVA12 strains and other EV-A, suggesting that there may be extensive genetic exchange between CVA12 and other enteroviruses. In summary, a total of 16 full-length CVA12 strains were reported in this study, providing valuable references for further studies of CVA12 worldwide.

## 1. Introduction

Enteroviruses belong to the genus Enterovirus of the *Picornaviridae* family and are currently composed of 12 enteroviruses (A-L) and 3 rhinoviruses (A-C) species (Zell et al., 2017). Human enteroviruses are grouped into HEV-A, HEV-B, HEV-C, and HEV-D, including over 100 serotypes of poliovirus, coxsackievirus, echovirus, and some newly discovered enteroviruses (King et al., 2018). Among them, the newly discovered enteroviruses currently have a total of 57 serotypes. The EV-A species currently consists of 25 serotype, of which Coxsackievirus A12 (CVA12) is a member. CVA12 is small, non-enveloped, single-stranded, and positive-sense RNA viruses. The genome is about 7.5 kb in length and contains a single long open reading frame (ORF) with a 5′-untranslated region (UTR) and a 3′-UTR on either side (Muslin et al., 2015, 2019). The polyprotein could be cleaved into P1, P2, and P3 protein precursors, followed by further cleavage into VP1-VP4, 2A-2C, and 3A-3D, respectively (Chen et al., 2020).

Due to cumulative mutations and/or recombination, enteroviruses have a high degree of genetic diversity and produce populations with related sequences (Muslin et al., 2019). The coding region of VP1 contains the most important antigen-specific neutralization sites of enterovirus (EV) capsid proteins, which is serotype specific (Song et al., 2020). Most genotyping of enterovirus is based on sequence analysis of VP1, such as EV-A71 (Mcwilliam Leitch et al., 2012), CVA16 (Zhang et al., 2010a), CVA6 (He et al., 2013), EV-B80 (Han et al., 2018), CVA2 (Yang et al., 2018) and CVB1 (Kim et al., 2013). Enteroviruses are the most common human pathogen (Zhao and Yang, 2021), and their spread either through the fecal-oral route or *via* respiratory transmission (Baggen et al., 2018; Wells and Coyne, 2019). From their primary replication sites in the gastrointestinal or respiratory tract, they can involve and infect other tissues and organs, including the central nervous system. They often infect children under 5 years of age and often cause large-scale infectious diseases such as HFMD, herpes angina, myocarditis, encephalitis, aseptic meningitis, acute flaccid paralysis (AFP) and acute flaccid myelitis, with fatal complications in severe cases (Brouwer et al., 2021; Zhao and Yang, 2021). CVA12 has been associated with different clinical symptoms, including AFP (Rao et al., 2020; Sousa et al., 2020), and has been isolated in many HFMD cases in recent years (Zhu et al., 2010; Wang et al., 2011; Liu et al., 2014; Puenpa et al., 2014; Guan et al., 2015; Timmermans et al., 2016). The prototype strain of CVA12 (Texas-12/AY421768.1) was first isolated in the United States in 1948, and no new CVA12 had been isolated for 55 years until it was isolated in Japan for the second time in 2003. After that, CVA12 has been increasingly isolated worldwide. Zhu et al. (2010) reported that CVA12 was associated with HFMD cases in Guangzhou city in 2008 (Guangdong Province, China). Liu et al. (2014) characterized the phylogeny of CVA12 for the first time based on the full-length VP1 sequence and whole genome sequences. The report revealed that Coxsackievirus A12 was one of the most common enteroviruses among non-EV71 and non-CVA16 enteroviruses associated with HFMD in Qingdao city (Shandong Province, China). In 2017–2018, Majumdar et al. (2021) detected 10 EV serotypes in Nigerian sewage for the first time, of which CVA12 was firstly described in Africa, although CVA12 was detected at a low frequency. Additionally, from 2009 to 2017, CVA12 was detected in specimens of HFMD cases reported in Jinan (Shandong Province), Yunnan Province and in Thailand (Puenpa et al., 2014; Guan et al., 2015; Rao et al., 2020).

According to previous reports, non-EV71 and non-CVA16 enteroviruses have gradually become important pathogens of HFMD in China, with CVA12 being one of the 12 serotype enteroviruses with the highest prevalence of HFMD in Jinan in 2009–2013 (Guan et al., 2015). However, other molecular epidemiological reports of CVA12 have only been found sporadically at home and abroad, and the global evolution and transmission history has not been systematically revealed. In this study, we contributed with 16 full-length genome sequences of CVA12 isolated from HFMD cases in 10 provinces of China between 2010 and 2019, deciphering the evolutionary dynamics and recombination patterns of CVA12 in mainland China.

## 2. Materials and methods

### 2.1. Virus isolation

From 2010 to 2019, 18,238 clinical samples of HFMD were obtained from all provinces in mainland China through the HFMD surveillance network established by our laboratory. First, the samples were standardized and the supernatant was extracted. The supernatant was inoculated into human rhabdomyosarcoma (RD) cells supplied by the American Center for Disease Control and Prevention for virus propagation and purification. Infected cell cultures were harvested after complete cytopathic effect was observed. Then, the genotypes of these harvested viruses were identified by PCR and sequencing. Finally, a total of 16 CVA12 strains were isolated (Supplementary Table S1). All these strains were once again inoculated into RD cells and cultured for the next study. All of these samples were collected from reports of sporadic mild HFMD cases in Shandong (*n* = 5), Tianjin (*n* = 2), Hebei (*n* = 2), Jiangxi (*n* = 1), Hainan (*n* = 1), Henan (*n* = 1), Shanxi (*n* = 1), Ningxia (*n* = 1), Chongqing (*n* = 1), and Zhejiang (*n* = 1).

### 2.2. CVA12 whole-genome sequencing

Viral RNAs were extracted from harvested 16 cell cultures using a QIAamp Viral RNA Mini Kit (Qiagen, Valencia, CA, United States) following the manufacturer’s protocol. Then, reverse transcription-polymerase chain reaction (RT-PCR) was performed to amplify the complete VP1 coding region (888 nt) using the PrimeScript One Step RT-PCR Kit Ver.2 (TaKaRa, Dalian, China) with previously designed primers (Liu et al., 2014). The primers used for PCR amplification and sequencing of the remaining genome in this study were designed based on the primer walking method (Table 1). The amplification program was as follows: 1 cycle of 30 min at 50°C, 1 cycle of 2 min at 94°C; 1 cycle of 30 s at 94°C; 40 cycles of 30 s at 50°C; and a final cycle of 1 min 20 s at 72°C. The PCR products were purified using a QIAquick PCR Purification Kit (Qiagen, Hilden, Germany), and then amplicons were bidirectionally sequenced using an ABI 3130 Genetic Analyzer (Applied Biosystems, Foster City, CA, United States). There is approximately 150–200 bp overlap among amplicons. Finally, the sequences obtained from different amplicons were assembled by the Sequencher program (Version 5.4.5) (GeneCodes, Ann Arbor, Michigan, United States) to harvest the whole genome sequence.

**Table 1:** Primers and names for amplifying the whole genome sequence of 16 CVA12 strains.

Primer	Position (nt)	Sequence (5′-3′)	Orientation
0001S48		GGGGACAAGTTTGTACAAAAAAGCA-GGCTTTAAAACAGCTCTGGGGTT	Forward
CVA12-943A	943–962	CCTCCGCACTAGGTGACTTC	Reverse
CVA12-526S	507–526	GTG GGTAGCGTGTCGTAATG	Forward
CVA12-1273A	1273–1292	TGCACTGCACGTGTATGCAG	Reverse
CVA12-890S	871–890	GGATTTCACCCAAGATCCAA	Forward
CVA12-1772A	1772–1791	CCAGGTAAAATGGGAGCTGA	Reverse
CVA12-1650S	1631–1650	GCCACAACAGCCATACCAAT	Forward
CVA12-2677A	2277–2296	CCAAGCCTGATCGAGAGAAG	Reverse
CVA12-2511S	2492–2511	ACCACTCAAACCCACCAAAC	Forward
CVA12-3599A	3599–3618	AGCATGAGGTGGGATTGGTA	Reverse
CVA12-3464S	3445–3464	GTCATCCACGACAGCTCAAG	Forward
CVA12-4536A	4536–4555	GTGGTCTGGGTCTGGAGGTA	Reverse
CVA12-3735S	3716–3735	GGACTCGTTGGCTTTGCTGA	Forward
CVA12-4744A	4744–4763	GTTGGTGGACGCGATGACAA	Reverse
CVA12-4462S	4443–4462	TCATCAGAGGCTCTCCAGGT	Forward
CVA12-5547A	5547–5566	CACCAATTCTACAGCGTCCA	Reverse
CVA12-4931S	4912–4931	CGCTGTAGTCCACTAGTGTG	Forward
CVA12-5921A	5921–5940	CTCTCCTTGTTCACTGGCGA	Reverse
CVA12-5405S	5384–5405	CCTCGATTTCGCTCTGTCTC	Forward
CVA12-6373A	6373–6392	TGGAGTAGGGAAGGTCCAAA	Reverse
CVA12-6213S	6194–6213	ATCGACACCTCCCAGATGAG	Forward
CVA12-7146A	7146–7165	GTCTTGAGTGTTGCGTGCAT	Reverse
CVA12-6884S	6865–6884	ATTCAAGGGCATTGATCTGG	Forward
7500A		GGGGACCACTTTGTACAAGAAAGCTGGG(T)^24^	Reverse

### 2.3. Dataset construction

In addition to the 16 strains from this study, we retrieved all CVA12 full-length VP1 sequences from the GenBank database by using “CVA12” and “Coxsackievirus A12” as search terms (dated April 2, 2021). To eliminate suspicious and low-quality sequences, we analyzed the regression of root tip distance to sampling time using TempEst V1.5.1 (Rambaut et al., 2016; Zhao et al., 2022). A total of 21 complete VP1 sequences were finally recruited from GenBank (Supplementary Table S2), all of which were from China except the prototype strain. We selected 37 full-length VP1 sequences for phylogenetic analysis and estimating the mean substitution rate and the time to most recent common ancestor (tMRCA) of CVA12. The phylogeographic analysis was performed using 36 full-length sequences. In addition, we obtained 17 EV-A prototype strains from GenBank.

### 2.4. Phylogenetic analysis

The phylogenetic characteristics were analyzed using 37 full-length VP1 nucleotide sequences. Sequence alignment was performed using the ClustalW tool in MEGA (version 7.0) (Sudhir Kumar, Arizona State University, Tempe, Arizona, United States). The best nucleotide substitution models “K2 + G + I” were selected by Modeltest (version 3.7). Then, the maximum likelihood (ML) method was used in the phylogenetic tree construction with 1,000 bootstrap replicates in MEGA7.

### 2.5. Phylodynamic analysis

In order to explore the evolutionary characteristics of CVA12, 37 full-length VP1 sequences were used for phylodynamic analysis. The presence of temporal signal was examined by root-to-tip regression with Tempest (version 1.5.3) software (Supplementary Figure S1). The Markov chain Monte Carlo (MCMC) method was performed in BEAST (version 1.10) and a strict clock model was used to estimate the temporal phylogeny and substitution rate of CVA12. The chain length was set to 80,000,000. Tracer (version 1.7.1) program was used to check for convergence. Effective sample size (ESS) >200 for all inferred parameters was accepted (Hu et al., 2021). A maximum clade credibility (MCC) tree was summarized using Tree Annotator (version 1.10.4), with the burn-in option used to remove the first 10% of sampled trees, and the resulting tree was visualized by FigTree (version 1.4.4). To estimate the effective population size of CVA12 circulating in mainland China, a Bayesian skyline plot was reconstructed using Tracer (version 1.7.1) program.

To understand the spatial dynamics of CVA12 in mainland China, 36 full-length VP1 sequences were selected for phylogeographic analyses to explore the possible migration patterns of CVA12. Phylogeographic analyses were performed in BEAST (version 1.10.4), using an asymmetric substitution model with BSSVS options to infer asymmetric diffusion rates between any pairwise location state, and allowing BF calculations to verify significant diffusion rates. Markov chain Monte Carlo (MCMC) sampling was performed in duplicate and samples were examined with Tracer (version1.7.1) to assess the convergence of parameters (ESS > 200; Mino et al., 2019). SpreaD3 software package (version 0.9.7) was used to analyze and visualize the pathogen phylodynamic reconstructions (Zhao et al., 2022). The BF value and the average posterior value (at least BF >3 and posterior mean value >0.5, the migration path is considered to be effective) can be used to infer the possible migration path between the two regions (Huang et al., 2020).

### 2.6. Recombination analysis

The neighbor-joining (NJ) phylogenetic trees of VP1, P1, P2 and P3 were constructed using the prototype EV-A strains in GenBank and the 16 CVA12 strains in this study. The recombination pattern was speculated based on the different positions and clustering of CVA12 strains on the phylogenetic tree. EV-A strains with high sequence homology with CVA12 recombinants in P2 and P3 regions were screened by BLAST, and full-length sequences with more than 85% similarity in GenBank were obtained as potential parents. Seven methods (RDP, GENECONV, MaxChi, Chimera, SiScan, Bootscan and 3Seq) were used to screen the recombinant signal in whole genome sequence by recombinant detection program 4 (RDP4, version 4.46). Then, SimPlot program (version 3.5.1) was used for similarity plots and bootscanning analysis to verify the recombination signal, with a 200-nucleotide window moving in 20-nucleotide steps.

### 2.7. Base substitution and amino acid mutation analysis

To further explore the differences between the 16 CVA12 strains and the CVA12 prototype strain, nucleotide and deduced amino acid sequences of 16 strains were compared with the CVA12 prototype strain using the BioEdit program (version 7.2.5.0). The antigenic sites of the coding region of the CVA12 prototype strain were predicted through the online website (IEDB.org: Free Epitope Database and Prediction Resource). In order to further study the antigenic site mutations of the 16 CVA12 strains, the amino acid sequences were compared between the 16 CVA12 strains and the CVA12 prototype strain.

## 3. Results

### 3.1. Full-length genomic characterization of the 16 CVA12 strains

Complete genome sequence of the 16 CVA12 strains were obtained using the primer walking strategy. The results showed that they were 7,391 to 7,403 nucleotides in length with a single ORF of 6,576 nucleotides encoding a single polypeptide of 2,191 amino acids. The sequences were flanked by a non-coding 5′-UTR of 735–745 nucleotides and a non-coding 3′-UTR of 81–83 nucleotides. The overall base composition of the 16 sequences was 27.41–27.64% of A, 23.40–24.12% of C, 24.16–24.48% of G, and 24.12–24.69% of *T*. The nucleotide and deduced amino acid sequences of the 16 sequences were compared with the CVA12 prototype strain (Table 2). The complete genome nucleotide sequence and amino acid identity among the 16 sequences was 89.2–99.7% and 97.2–100%, respectively. Furthermore, the 16 sequences showed 78.8–79% nucleotide identity and 94–94.2% amino acid identity with the CVA12 prototype strain.

**Table 2:** Pairwise comparisons of nucleotide sequences and amino acid sequences among the 16 CVA12 strains and with the CVA12 prototype strain (AY421768.1/Texas-12).

Nucleotide identity (%) [amino identity(%)]													
Strain name	5′-UTR	P1				P2			P3				3′-UTR
		VP4	VP3	VP2	VP1	2A	2B	2C	3A	3B	3C	3D	
CHN/CQ/2013/70	81.7	77.2	81.2	80.3	81.6	78.8	78.1	80.7	76.7	71.2	78.1	78.4	79.5
		[89.8]	[95.8]	[95.6]	[95.6]	[94]	[94.9]	[96.9]	[95.3]	[90.9]	[95]	[93]	
CHN/HAN/2017/17	82.5	77.2	80.6	80.2	80.4	78.4	77.4	80.7	79	71.2	76.3	78	79.5
		[91.3]	[96.6]	[96]	[94.5]	[94]	[93.9]	[96.3]	[95.3]	[90.9]	[94.5]	[92.6]	
CHN/HB/2015/64	82.1	77.2	81.1	80.9	81.9	78.2	77.7	81.2	76.7	71.2	76.6	78.3	80.7
		[89.8]	[96.2]	[96]	[95.2]	[94]	[93.9]	[96.9]	[95.3]	[90.9]	[93.9]	[93]	
CHN/HB/2016/16	80.8	77.7	80.4	80.2	81.3	78.2	77.7	80.7	77.1	71.2	77.2	78.1	78.3
		[89.8]	[96.2]	[96]	[95.6]	[94]	[93.9]	[96.9]	[95.3]	[90.9]	[95]	[93]	
CHN/HN/2019/214	82.4	76.3	80.5	80.1	80.9	77.5	77.7	79.2	79	72.7	77.9	77.7	78.3
		[89.8]	[95.8]	[95.6]	[94.5]	[92.6]	[92.9]	[97.5]	[97.6]	[90.9]	[94.5]	[93.2]	
CHN/JX/2013/40	80.6	77.2	81.2	80.5	81.3	78.8	78.4	80.5	75.9	71.2	77.7	78.3	78.3
		[88.4]	[96.2]	[96]	[94.5]	[94]	[94.9]	[96.9]	[95.3]	[90.9]	[95]	[93]	
CHN/NX/2010/83	82.3	77.7	81.3	81.1	80.9	78.6	78.7	80.2	77.5	72.7	76.8	77.9	78.3
		[89.8]	[96.2]	[96]	[94.9]	[95.3]	[94.9]	[97.2]	[95.3]	[86.3]	[95]	[93]	
CHN/SD/2015/72	81.7	77.2	80.8	80.7	82	78.6	77.7	81	76.7	71.2	77	78.2	80.7
		[89.8]	[96.2]	[96]	[95.6]	[94]	[93.9]	[96.9]	[95.3]	[90.9]	[95]	[93]	
CHN/SD/2015/73	81.6	77.2	80.6	80.7	82	78.4	77.7	80.9	77.1	71.2	77	78.2	80.7
		[89.8]	[96.2]	[96]	[95.6]	[94]	[93.9]	[96.9]	[95.3]	[90.9]	[95]	[93]	
CHN/SD/2015/81	81.2	75.3	80.2	80.1	81.4	78.4	76.4	80.4	75.5	74.2	76.8	77.7	79.5
		[89.8]	[95.8]	[95.6]	[95.6]	[94]	[92.9]	[96.9]	[93]	[90.9]	[95]	[92.8]	
CHN/SD/2016/71	81.6	76.8	80.5	80.6	80.8	78.2	78.1	80.6	76.7	71.2	77	77.9	78.3
		[89.8]	[96.2]	[96]	[95.6]	[94]	[93.9]	[96.9]	[95.3]	[90.9]	[95]	[93]	
CHN/SD/2016/72	82.7	76.8	80.5	80.5	80.8	78.4	78.4	80.6	77.1	71.2	77.2	77.7	79.5
		[89.8]	[96.2]	[96]	[95.6]	[94]	[94.9]	[96.9]	[95.3]	[90.9]	[95.6]	[93]	
CHN/SX/2011/31	81.5	78.2	81.6	80.3	80.9	78.8	77.1	79.5	77.9	71.2	77.4	77.6	78.3
		[89.8]	[96.2]	[95.6]	[94.5]	[95.3]	[93.9]	[96.3]	[94.1]	[90.9]	[95]	[92.4]	
CHN/TJ/2015/92	82	77.2	81.2	80.6	81.5	78	77.7	80.6	77.1	69.6	77.5	77.9	72.0
		[89.8]	[96.2]	[96]	[94.9]	[94]	[93.9]	[96.9]	[95.3]	[90.9]	[95]	[92.8]	
CHN/TJ/2016/97	82.5	75.8	80.5	80.1	80.4	78	77.7	80.3	77.1	71.2	76.8	77.9	79.5
		[89.8]	[96.2]	[96]	[94.9]	[93.3]	[92.9]	[96.9]	[95.3]	[90.9]	[95]	[93]	
CHN/ZJ/2017/69	81	75.3	80.1	80	80.8	79.1	78.7	80.6	76.7	71.2	77	77.8	79.5
		[89.8]	[96.2]	[95.6]	[95.2]	[94]	[93.9]	[96.6]	[95.3]	[90.9]	[95]	[93]	

### 3.2. Phylogenetic analysis

**Figure 1:**
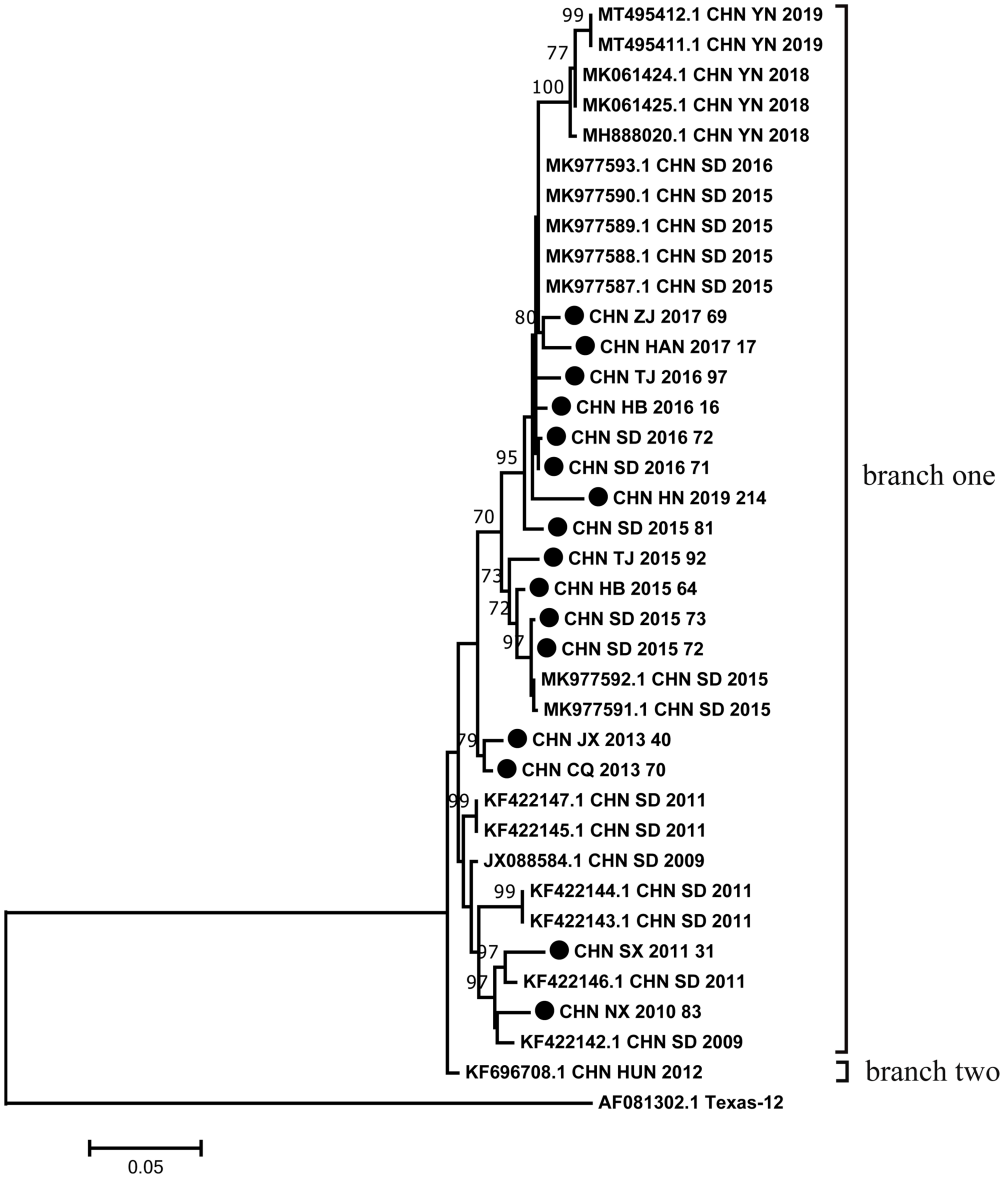
Phylogenetic analysis based on the full-length VP1 sequences of 16 CVA12 from this study and 21 sequences retrieved from the GenBank. Bootstrap values greater than 70% were highlighted. The 16 CVA12 strains from this study are represented by black dots.

The phylogenetic tree was constructed with maximum likelihood method based on VP1 region of 16 sequences from this study and 21 sequences retrieved from the GenBank. Phylogenetic analysis based on the full-length VP1 region showed that all CVA12 strains could be divided into two branches except for the prototype strain (Figure 1). The vast majority of CVA12 strains clustered in branch one, while CHN_HUN_2012 strain was divided into a separate branch. The pairwise distance (p-distance) between two branches was 3.8%, and they differed significantly from the prototype strain with p-distance, reaching 25.8 and 27.1%, respectively. The phylogenetic tree showed that the 16 strains which collected from different provinces in mainland China were clustered in branch one with scattered distribution. Moreover, some sequences from different provinces were clustered together. For example, CHN_JX_2013_40 strain was clustered with CHN_CQ_2013_70 strain. The results of phylogenetic analysis revealed that CVA12 has been circulating in mainland China till present, which involves several provinces, showing a continuous evolutionary trend over time.

### 3.3. Phylodynamic analysis

**Figure 2:**
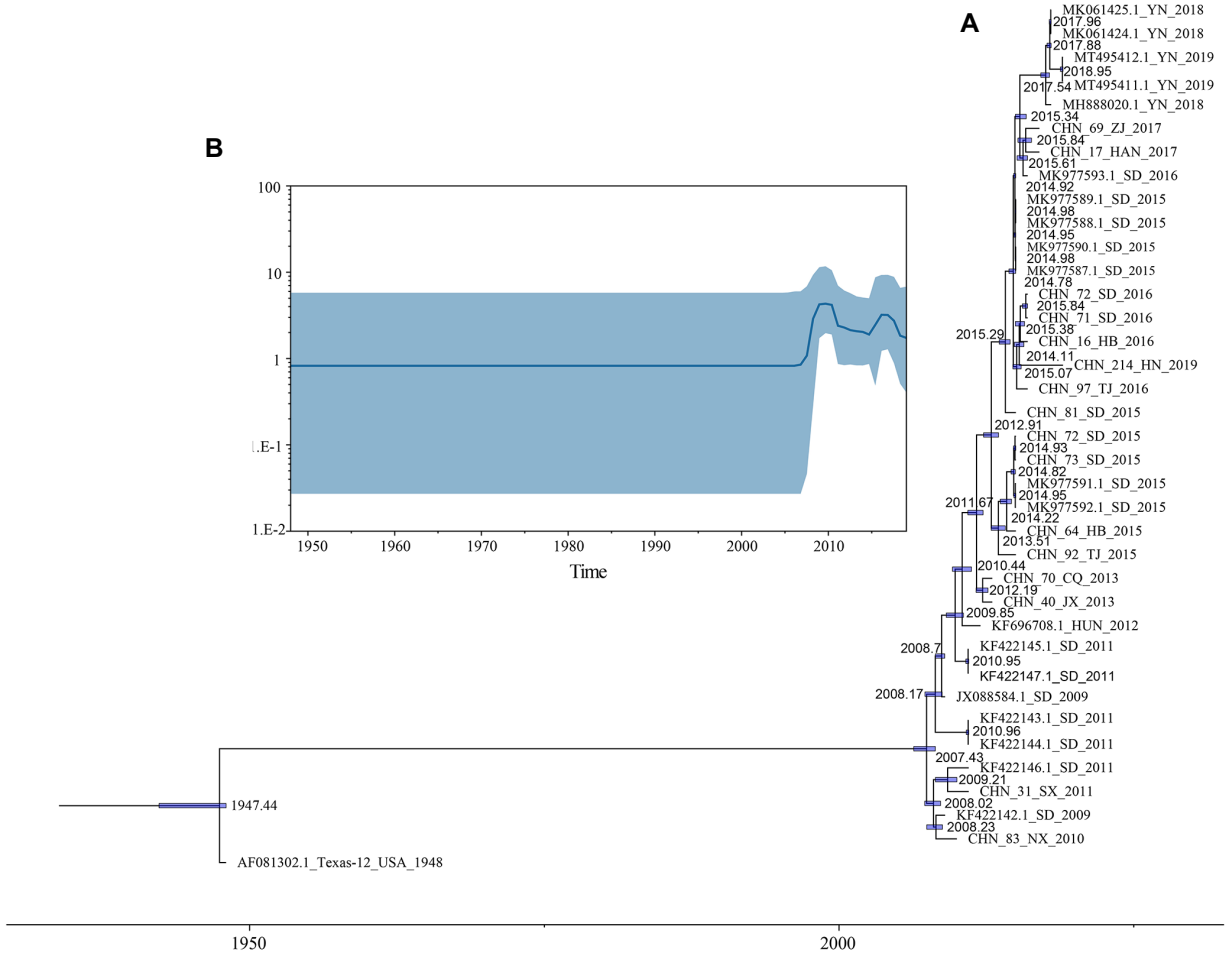
**(A)** A phylogenetic tree with Maximum Clade Credibility (MCC) was constructed based on the full-length VP1 sequences of CVA12 in GenBank and 16 CVA12 sequences from this study. The blue bar represents 95% HPDs of tMRCAs. **(B)** A Bayesian skyline plot based on the full-length VP1 sequences of CVA12 reflects the effective population size in mainland China from 2009 to 2019.

Based on 37 full-length VP1 sequences, MCMC method and strict clock model were used to generate MCC tree and estimate the substitution rate (Figure 2A). The computed MCC tree was similar to the phylogenetic trees in topology, showing that CVA12 has been in continuous evolution. The substitution rate for the VP1 region was 6.61 × 10^−3^ (95% HPD range 5.16–8.20 × 10^−3^) substitution/site/year, with a predicted time to most recent common ancestor (tMRCA) of 1946 (95% HPD: 1942–1947). The MCC tree showed that the earliest node date of CVA12 in China was 2007. We analyzed the population dynamics of CVA12 using Bayesian skyline. The results revealed that the effective population size remained unchanged from 1948 to 2007. After 2007, with the increase in the number of CVA12 strains, the effective population size fluctuated twice, which occurred from 2009 to 2010 and 2015 to 2016, respectively. From 2018 to 2019, it gradually decreased, but still remained at a relatively high level (Figure 2B).

**Figure 3:**
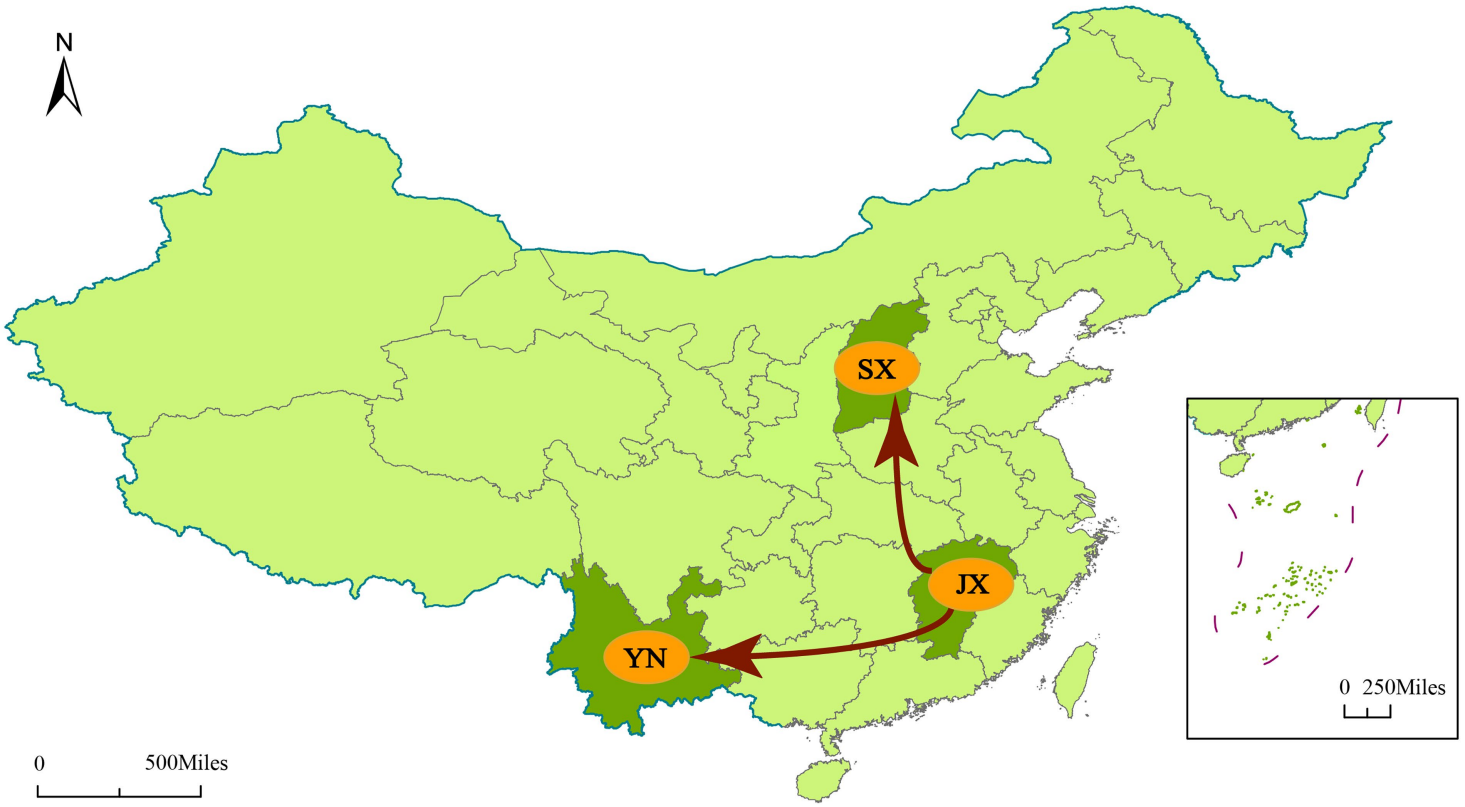
Spatial spread links of CVA12 between sampling locations in Mainland China. The figure shows only the state transitions with supported BF ≥ 3 and posterior mean value ≥0.5. Red arrow, Very strongly supported rates with BF ≥ 100.

To understand the transmission path of CVA12 in mainland China, we reconstructed the spatial transmission patterns from 2009 to 2019 based on 36 complete VP1 sequences from 12 provinces (that is, the Shandong, Tianjin, Henan, Hebei, Shanxi, Hunan, Zhejiang, Jiangxi, Yunnan, Hainan, Ningxia and Chongqing; Figure 3). Phylogeographic analysis indicated that CVA12 strains probably originated from Jiangxi province. There are two significant migration pathways of inter-provincial transmission: (1) Jiangxi to Shanxi; (2) Jiangxi to Yunnan (with BF very strongly supported ≥100; Supplementary Table S3).

### 3.4. Recombination analysis

**Figure 4:**
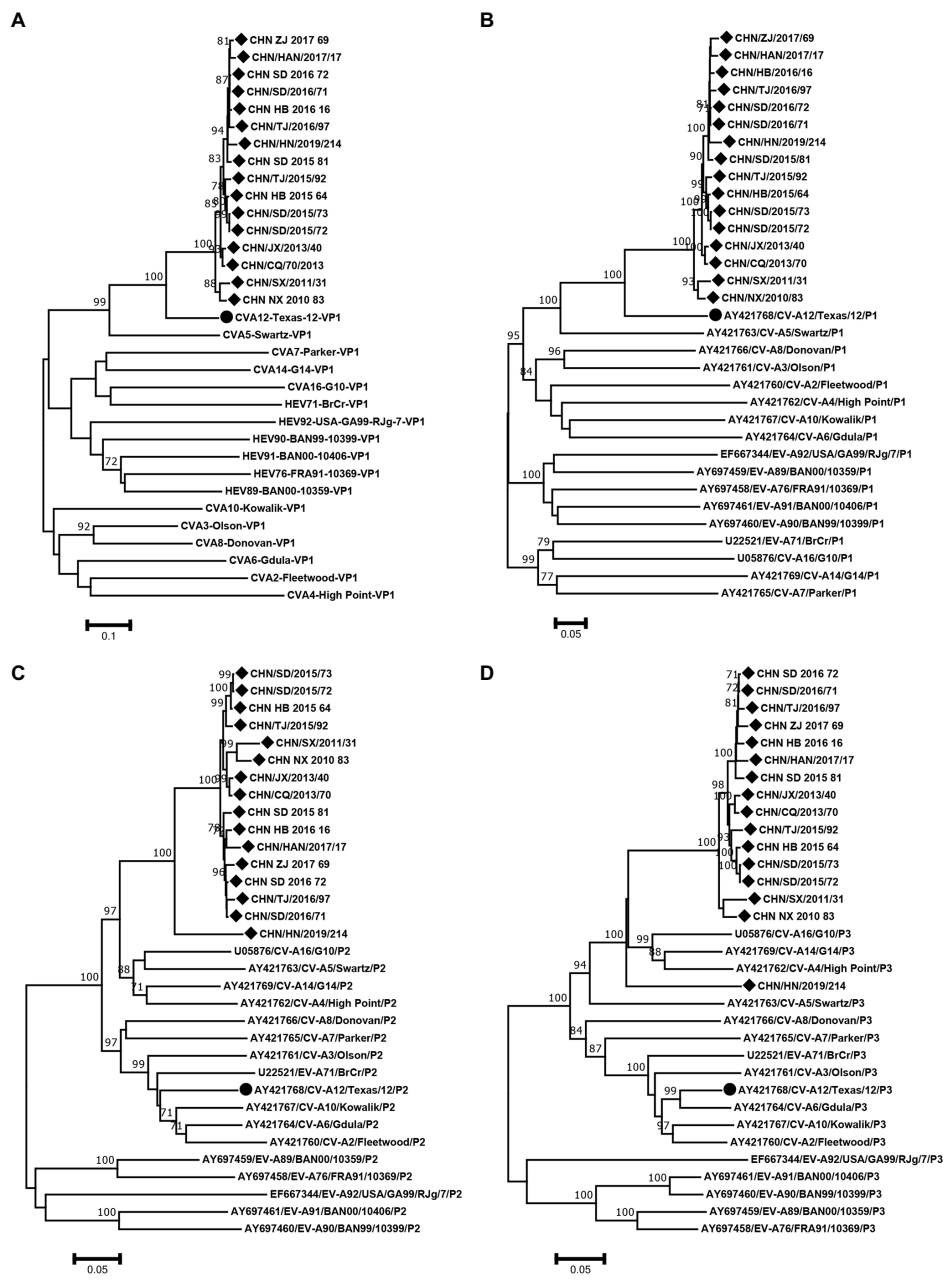
Neighbor-joining phylogenetic trees of **(A)** VP1, **(B)** P1, **(C)** P2 and **(D)** P3 were constructed based on 16 CVA12 strains from this study and EV-A prototype strains. The 16 CVA12 strains from this study are represented by a diamond, and CVA12 prototype strain is represented by a circle. The numbers on these nodes indicate the bootstrap support for that node (the percentage of 1000 bootstrap replicates). The scale bars indicate the genetic distance.

The phylogenetic trees of VP1, P1, P2 and P3 were constructed based on the EV-A prototype strains in the GenBank and 16 sequences from this study. The results of VP1 and P1 phylogenetic trees showed that the 16 sequences were clustered with CVA12 prototype strain, as expected (Figures 4A,B). However, the phylogenetic trees constructed based on the P2 and P3 coding regions showed different results. In the P2 region, the 16 sequences were closest to CVA16, CVA5, CVA14 and CVA4 (Figure 4C). In the P3 region, they were closest to CVA16, CVA14 and CVA4 (Figure 4D). It is noteworthy that CHN_HN_2019_214 strain was far from the other CVA12 strains in the P3 region.

RDP4 was used to analyze the recombination signal. At least three of the seven methods detected recombination, and the *p* value cutoff was 0.05, which was considered to be true recombination (Martin et al., 2017). The results showed that there were two different recombination patterns between CHN_HN_2019_214 strain and other 15 strains. CHN_SD_2015_81 strain was randomly selected from 15 strains for further analysis. The breakpoint positions of CHN_SD_2015_81 strain were mainly located in 65–424, 3406–5437 and 5438–7476, covering part or all the 5′-UTR, 2A-3′-UTR regions of the genome. The major parent was CVA12 prototype strain (GenBank number: AY421768.1). The minor parents were EV71 (GenBank number: MT241237.1, DQ341354.1 and KP289419.1). Recombination events were detected in CHN_HN_2019_214 strain at positions 22–94 and 3573–4126. The major parents were CVA2 (GenBank number: KX595284.1 and MK967658.1), and the minor parent was unknown.

**Figure 5:**
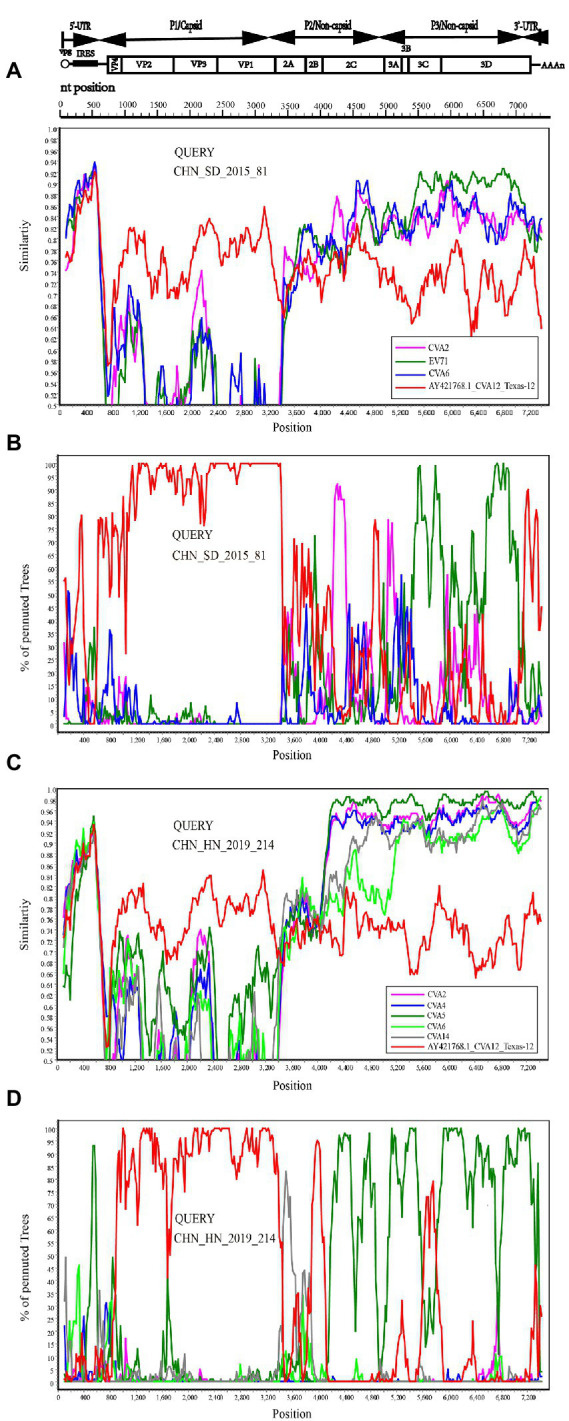
Recombination events of CHN_SD_2015_81 and CHN_HN_2019_214 strains were investigated based on Similarity plots and bootscanning analyses. Panels **(A,C)** are the similarity diagram, and Panels **(B,D)** are the guided scan analysis. CHN_SD_2015_81 and CHN_HN_2019_214 strains were used as query sequences (shown in black font).

To confirm the recombination events between the CHN_SD_2015_81, CHN_HN_2019_214 strains and other EV-A strains, similarity plots and bootscanning analyses were performed. CHN_SD_2015_81 and CHN_HN_2019_214 strains were picked as query sequences. In the P1 coding region, CHN_SD_2015_81 and CHN_HN_2019_214 strains showed the highest similarity with the CVA12 prototype strain, as expected. However, in the other regions, the results were significantly different. CHN_SD_2015_81 strain and CVA2 group showed a narrow range of recombination in the P2 region when CHN_SD_2015_81 strain was used as the query sequence. In P3 regions, CHN_SD_2015_81 strain and EV-71 group had extensive recombination (Figures 5A,B). When CHN_HN_2019_214 strain was used as the query sequence, CHN_HN_2019_214 strain had small fragment recombination with CVA5 group in the 5’-UTR region. In the P2 and P3 regions, CHN_HN_2019_214 strain had extensive recombination with CVA5 group (Figures 5C,D). These data complemented and validated the recombinant results predicted by P2, P3 phylogenetic trees and RDP4. These results showed that recombination events occurred in the non-capsid region of the 16 strains, and CHN_HN_2019_214 strain had different recombination events compared with the other 15 strains.

### 3.5. Base substitution and amino acid mutation analysis

Enteroviruses have a high mutation rate due to the lack of proofreading activity during genome replication (Santti et al., 2000). Compared with the CVA12 prototype strain, the genome of 16 sequences contained 1495–1536 nucleotide mutations and 108–118 amino acid mutations. The amino acid mutations contained 74 common mutation sites, in which 9 non-polar amino acids were replaced by polar amino acids and 8 polar amino acids were replaced by non-polar amino acids. In addition, 16 CVA12 sequences had a nucleotide deletion at sites 109, 129, 689, 7339, 7340 and a nucleotide insertion at site 714. The nucleotide and amino acid differences between the 16 sequences were 21–793 and 0–60, respectively. As there is no relevant report on the antigenic sites of CVA12 at present, we predicted the possible antigenic sites of CVA12 through the online website, providing reference for the next study of CVA12. The results showed that there were 8–10 antigenic sites in the VP1-VP3 region of the CVA12 prototype strain, respectively (Table 3). Comparing the differences of antigen sites between the 16 sequences and CVA12 prototype strain, 13 common antigen mutation sites were found: [VP1: S→N[17], T→N[35], N→S[60], R→K[71], A→T[290]; VP2: N→S[74], E→T[148], T→V[151], H→Q[159], D→E[160]); VP3: T→S[10], T→V[20], M→N[33], (Pfeiffer and Kirkegaard, 2005; Zhu et al., 2010; Gnadig et al., 2012; Han et al., 2018; Rao et al., 2020). It has been reported that mutation is one of the main ways of enterovirus evolution, and the extent of this mutant swarm is crucial to the viral adaptability, dissemination, and pathogenesis (Pfeiffer and Kirkegaard, 2005; Lauring and Andino, 2010; Gnadig et al., 2012). Therefore, further virological studies are necessary to understand the role of these mutations in the evolution of CVA12.

**Table 3:** The antigen sites of CVA12 prototype strain (AY421768.1/Texas-12) were predicted through online websites (IEDB.org: Free Epitope Database and Prediction Resource).

Region	Position	Antigen epitope
VP1	17–77	SAISTTQTHQTAADTRVSTHRLGTGEVPALQAAETGATSNATDENMIETRCVVNRHGVSET
	95–105	EDATATNKGYA
	108–115	EIDVMGFA
	140–144	RNGST
	159–172	PVPTGRDTFQWQSA
	207–220	PTFGERPVTTNMNY
	239–242	EASG
	265–292	RSQLYLLKNYPNFDNTKILNASHNRASI
VP2	6–19	ACGYSDRVAQLTVG
	21–24	STIT
	26–27	QE
	39–60	PEYCADTDATAVDKPTRPDVSV
	70–77	MWDKNSKG
	83–88	PDLLTQ
	132–161	VAGMGPGDKPQNAPHPEYQTTQPGAGGHDL
	204–209	YDSAIH
	225–231	YDTGATT
	247–251	GGLRQ
VP3	5–39	ELKPGTNQFLTTDDEISAPILPGFNATPMIHIPGE
	41–42	TN
	56–64	NNVTNVDGM
	75–77	TEA
	89–95	GRDGPWE
	137–148	GGQQPATRDIAM
	175–185	YRTVETGGILD
	202–205	VPNG
	229–237	DTESISQTA

## 4. Discussion

From 2009 to 2021, the number of HFMD cases in mainland China continued to top the list of notifiable infectious diseases except for 2019 and 2020. Before 2018, EV-A71 was the main cause of severe cases and deaths, while other enteroviruses were the main cause between 2019 and 2021. Although EV-A71 and CVA16 are generally regarded as major pathogens of HFMD globally (Chen et al., 2010; Wu et al., 2010; Zhang et al., 2010a, 2013; Han et al., 2019; Zhang et al., 2010b), an increasing number of other enteroviruses, including CVA12, have been isolated from HFMD cases (Guan et al., 2015).

According to Liu et al., (2014), for HFMD not associated with EV-A71 or CVA16, CVA12 was one of the most common enteroviruses in the HFMD cases in Qingdao. In this study, the phylogenetic tree constructed based on the full-length VP1 sequence was divided into two branches. In terms of p-distance between branches, the two branches differed significantly from the prototype strain. These differences suggested that CVA12 strains have been evolving, although no new CVA12 was isolated in mainland China between 1948 and 2008. CHN_HUN_2012 strain was divided into a separate branch, suggesting that its evolutionary trend was different from other strains and may develop into a new lineage. The prototype strain CVA12 was isolated from the United States in 1948 and was an extinct genetic lineage. The phylogenetic tree showed that the 16 strains were scattered distribution. Moreover, some sequences from different provinces were clustered together. These results indicated that there may be inter-provincial transmission of CVA12 in mainland China with multiple transmission chains. The above data revealed that CVA12 has been in a state of mutual diffusion and continuous differentiation since its discovery in mainland China. Therefore, CVA12 may be associated with HFMD outbreaks and evolve as a major pathogen. Notably, we used Sanger sequencing techniques, which could not identify the fold coverage for each genome. In the future, the application of next-generation sequencing techniques in the monitoring of CVA12 may better explore their genetic characteristics.

As RNA viruses, Enteroviruses have great genetic variability and rely on two different evolutionary mechanisms of mutation and recombination in order to be able to quickly respond and adapt to new environment. Therefore, mutation and recombination are the main driving force of enterovirus evolution and genetic architecture shaping of enteroviruses (Han et al., 2018; Muslin et al., 2019). A previous study (Khan and Khan, 2021) showed that samples from CVA12 and CVA6 genotypes exhibited genetic stratification due to existence of admixed or recombinant strains. This may provoke the emergence of new lineages within the CVA12 and CVA6 enterovirus serotypes with a distinctive infection burden. CHN_SD_2015_81 and CHN_HN_2019_214 strains were far from the prototype strain in the phylogenetic tree of P2 and P3 regions, suggesting that there may be recombination in the P2 and P3 coding regions. CHN_HN_2019_214 strain was far away from other isolates in the P3 region, suggesting that CHN_HN_2019_214 strain may have undergone different recombination events during evolution. RDP4 and Simplot confirmed the presence of recombination events in 16 CVA12 strains. The high mutation and two different recombination patterns may lead to genetic variation between CVA12 and other enterovirus species. This leads to the emergence of lineages with altered characteristics. In addition, recombination can generate chimeric molecules from parental genomes of different phylogenetic origins and may also help enteroviruses to acquire combined advantageous features from various genomes during the process of evolution (Lukashev et al., 2004; Bouslama et al., 2007; Zhang et al., 2015; Song et al., 2020). This may prompt the evolution of CVA12 into a new recombinant with higher virulence and transmissibility. Although few CVA12 strains have been isolated from HFMD, the potential mutations and recombination make it likely to be an important pathogen of HFMD.

Phylogeographic analysis indicated that CVA12 had been spreading in mainland China from 2009 to 2019, and there were two significant migration pathways. This is consistent with the results of phylogenetic and recombination analyses. However, due to incomplete surveillance of enteroviruses and the lack of studies on CVA12, the number of full-length VP1 sequences of CVA12 available in GenBank is very limited. This study can only reflect the evolutionary characteristics of CVA12 in mainland China, and the global evolutionary characteristics need to be further studied. Moreover, the data may have created some bias in the results due to the sample size and temporal and spatial difference. However, we tried to collect all the full-length VP1 sequences of CVA12 from GenBank to explore the possible origin time and substitution rate. The full-length VP1 sequences of CVA12 in GenBank were derived from China except for the prototype strain. The reason may be the prevalence of CVA12 in China. Another possible reason is that improved enterovirus surveillance in mainland China has led to the discovery of more CVA12 strains. Therefore, global surveillance of enteroviruses should also be strengthened. The genetic characteristics and evolutionary rules of enteroviruses should be adequately studied to prevent large-scale outbreaks of diseases such as HFMD.

In conclusion, we reported the full-length genome sequences of 16 CVA12 strains from mainland China from 2010 to 2019. The high difference between CVA12 strains and the CVA12 prototype strain indicated that these viruses could have been circulating in mainland China for many years, although CVA12 was not reported before 2008. Based on the results of phylogenetic and recombination analyses, we speculated that mutations and recombination with other enteroviruses may be the main evolutionary mode of CVA12, which may lead to changes in virulence and transmissibility, and induce associated diseases. At the same time, we estimated the evolutionary origin and substitution rate of CVA12 using phylodynamic analysis, and discovered two significant migration pathways of CVA12 in mainland China (very strongly support with BF ≥100). This study has deepened our understanding of CVA12 and provided valuable information on the molecular epidemiology of CVA12.

## Data availability statement

The datasets presented in this study can be found in online repositories. The names of the repository/repositories and accession number(s) can be found in the article/Supplementary material.

## Author contributions

DY, YZ, and WX: conceptualization. QG, HZ, HL, and JX: methodology. QG: software, formal analysis, investigation, data curation, writing – original draft preparation, and visualization. QG and QiaY: validation. TJ, XW, QiuY, ZT, SZ and DW: resources. DY: writing – review and editing, and supervision. YZ: project administration. YZ and DY: funding acquisition. All authors have read and agreed to the published version of the manuscript.

## Funding

This study was supported by grants from the National Key R&D (Project No. 2021YFF0703801), the National Key Research and Development Program of China (Project No. 2021YFC2302003), and the Natural Science Foundation of Beijing (Project No. L192014).

## Conflict of interest

The authors declare that the research was conducted in the absence of any commercial or financial relationships that could be construed as a potential conflict of interest.

## Publisher’s note

All claims expressed in this article are solely those of the authors and do not necessarily represent those of their affiliated organizations, or those of the publisher, the editors and the reviewers. Any product that may be evaluated in this article, or claim that may be made by its manufacturer, is not guaranteed or endorsed by the publisher.

## References

[ref1] Baggen J. Thibaut H. J. Strating J. van Kuppeveld F. J. M. (2018). The life cycle of non-polio enteroviruses and how to target it. Nat. Rev. Microbiol. 16, 368–381. doi: 10.1038/s41579-018-0005-4, PMID: 29626210

[ref2] Bouslama L. Nasri D. Chollet L. Belguith K. Bourlet T. Aouni M. (2007). Natural recombination event within the capsid genomic region leading to a chimeric strain of human enterovirus B. J. Virol. 81, 8944–8952. doi: 10.1128/JVI.00180-07, PMID: 17537864 PMC1951430

[ref3] Brouwer L. Moreni G. Wolthers K. C. Pajkrt D. (2021). World-wide prevalence and genotype distribution of enteroviruses. Viruses 13:434. doi: 10.3390/v13030434, PMID: 33800518 PMC7999254

[ref4] Chen J. Han Z. Wu H. Xu W. Yu D. Zhang Y. (2020). A large-scale outbreak of echovirus 30 in Gansu Province of China in 2015 and its phylodynamic characterization. Front. Microbiol. 11:1137. 32587581 10.3389/fmicb.2020.01137 PMC7297909

[ref5] Chen S. Huang Y. Li W. Chiu C.-H. Huang C.-G. Tsao K.-C. (2010). Comparison of clinical features between coxsackievirus A2 and enterovirus 71 during the enterovirus outbreak in Taiwan, 2008: a children's hospital experience. J. Microbiol. Immunol. Infect. 43, 99–104. doi: 10.1016/S1684-1182(10)60016-3, PMID: 20457425

[ref6] Gnadig N. F. Beaucourt S. Campagnola G. Bordería A. V. Sanz-Ramos M. Gong P. (2012). Coxsackievirus B3 mutator strains are attenuated in vivo. Proc. Natl. Acad. Sci. U. S. A. 109, E2294–E2303. doi: 10.1073/pnas.1204022109, PMID: 22853955 PMC3427060

[ref7] Guan H. Wang J. Wang C. Yang M. Liu L. Yang G. (2015). Etiology of multiple non-EV71 and non-CVA16 enteroviruses associated with hand, foot and mouth disease in Jinan, China, 2009-June 2013. PLoS One 10:e0142733. doi: 10.1371/journal.pone.0142733, PMID: 26562154 PMC4642993

[ref8] Han Z. Zhang Y. Huang K. Cui H. Hong M. Tang H. (2018). Genetic characterization and molecular epidemiological analysis of novel enterovirus EV-B80 in China. Emerg. Microbes Infect. 7:193. doi: 10.1038/s41426-018-0196-9, PMID: 30482903 PMC6258725

[ref9] Han Z. Zhang Y. Huang K. Wang J. Tian H. Song Y. (2019). Two Coxsackievirus B3 outbreaks associated with hand, foot, and mouth disease in China and the evolutionary history worldwide. BMC Infect. Dis. 19:466. doi: 10.1186/s12879-019-4107-z, PMID: 31126252 PMC6534883

[ref10] He Y. Q. Chen L. Xu W. B. Yang H. Wang H. Z. Zong W. P. (2013). Emergence, circulation, and spatiotemporal phylogenetic analysis of coxsackievirus a6-and coxsackievirus a10-associated hand, foot, and mouth disease infections from 2008 to 2012 in Shenzhen, China. J. Clin. Microbiol. 51, 3560–3566. doi: 10.1128/JCM.01231-13, PMID: 23966496 PMC3889734

[ref11] Hu Y. F. Jia L. P. Yu F. Y. Liu L. Y. Song Q. W. Dong H. J. (2021). Molecular epidemiology of coxsackievirus A16 circulating in children in Beijing, China from 2010 to 2019. World J. Pediatr. 17, 508–516. doi: 10.1007/s12519-021-00451-y, PMID: 34453285 PMC8523403

[ref12] Huang K. Zhang Y. Han Z. Zhou X. Song Y. Wang D. (2020). Global spread of the B5 subgenotype EV-A71 and the Phylogeographical analysis of Chinese migration events. Front. Cell. Infect. Microbiol. 10:475. doi: 10.3389/fcimb.2020.00475, PMID: 33102246 PMC7546772

[ref13] Khan H. Khan A. (2021). Genome-wide population structure inferences of human coxsackievirus-a; insights the genotypes diversity and evolution. Infect. Genet. Evol. 95:105068. doi: 10.1016/j.meegid.2021.105068, PMID: 34492386

[ref14] Kim H. Kang B. Hwang S. Hong J. Chung J. Kim S. (2013). Molecular characteristics of human coxsackievirus B1 infection in Korea, 2008-2009. J. Med. Virol. 85, 110–115. doi: 10.1002/jmv.23359, PMID: 23073968

[ref15] King A. M. Q. Lefkowitz E. J. Mushegian A. R. Adams M. J. Dutilh B. E. Gorbalenya A. E. (2018). Changes to taxonomy and the international code of virus classification and nomenclature ratified by the international committee on taxonomy of viruses (2018). Arch. Virol. 163, 2601–2631. doi: 10.1007/s00705-018-3847-1, PMID: 29754305

[ref16] Lauring A. S. Andino R. (2010). Quasispecies theory and the behavior of RNA viruses. PLoS Pathog. 6:e1001005. doi: 10.1371/journal.ppat.1001005, PMID: 20661479 PMC2908548

[ref17] Liu X. Mao N. Yu W. Chai Q. Wang H. Wang W. (2014). Genetic characterization of emerging coxsackievirus A12 associated with hand, foot and mouth disease in Qingdao, China. Arch. Virol. 159, 2497–2502. doi: 10.1007/s00705-014-2067-6, PMID: 24796551

[ref18] Lukashev A. N. Lashkevich V. A. Koroleva G. A. Ilonen J. Hinkkanen A. E. (2004). Recombination in uveitis-causing enterovirus strains. J. Gen. Virol. 85, 463–470. doi: 10.1099/vir.0.19469-0, PMID: 14769904

[ref19] Majumdar M. Klapsa D. Wilton T. Bujaki E. Fernandez-Garcia M. D. Faleye T. O. C. (2021). High diversity of human non-polio enterovirus serotypes identified in contaminated water in Nigeria. Viruses 13:249. doi: 10.3390/v13020249, PMID: 33562806 PMC7914538

[ref20] Martin D. P. Murrell B. Khoosal A. Muhire B. (2017). Detecting and analyzing genetic recombination using RDP4. Methods Mol. Biol. 1525, 433–460. doi: 10.1007/978-1-4939-6622-6_17, PMID: 27896731

[ref21] Mcwilliam Leitch E. C. Cabrerizo M. Cardosa J. Harvala H. Ivanova O. E. Koike S. (2012). The association of recombination events in the founding and emergence of subgenogroup evolutionary lineages of human enterovirus 71. J. Virol. 86, 2676–2685. doi: 10.1128/JVI.06065-11, PMID: 22205739 PMC3302253

[ref22] Mino S. Mojsiejczuk L. Guo W. Zhang H. Qi T. Du C. (2019). Equine influenza virus in Asia: phylogeographic pattern and molecular features reveal circulation of an autochthonous lineage. J. Virol. 93:e00116. doi: 10.1128/JVI.00116-19, PMID: 31019053 PMC6580976

[ref23] Muslin C. Joffret M. L. Pelletier I. Blondel B. Delpeyroux F. (2015). Evolution and emergence of enteroviruses through intra-and inter-species recombination: plasticity and phenotypic impact of modular genetic exchanges in the 5' Untranslated region. PLoS Pathog. 11:e1005266. doi: 10.1371/journal.ppat.1005266, PMID: 26562151 PMC4643034

[ref24] Muslin C. Mac K. A. I. N. A. Bessaud M. Blondel B. Delpeyroux F. (2019). Recombination in enteroviruses, a multi-step modular evolutionary process. Viruses 11:859. doi: 10.3390/v11090859, PMID: 31540135 PMC6784155

[ref25] Pfeiffer J. K. Kirkegaard K. (2005). Increased fidelity reduces poliovirus fitness and virulence under selective pressure in mice. PLoS Pathog. 1:e11. doi: 10.1371/journal.ppat.0010011, PMID: 16220146 PMC1250929

[ref26] Puenpa J. Mauleekoonphairoj J. Linsuwanon P. Suwannakarn K. Chieochansin T. Korkong S. (2014). Prevalence and characterization of enterovirus infections among pediatric patients with hand foot mouth disease, herpangina and influenza like illness in Thailand, 2012. PLoS One 9:e98888. doi: 10.1371/journal.pone.0098888, PMID: 24887237 PMC4041783

[ref27] Rambaut A. Lam T. T. Max Carvalho L. Pybus O. G. (2016). Exploring the temporal structure of heterochronous sequences using TempEst (formerly path-O-gen). Virus Evol. 2:vew007. doi: 10.1093/ve/vew007, PMID: 27774300 PMC4989882

[ref28] Rao Q. Zhang Z. Jiang H. Wang M. Huang R. Du T. (2020). Comparison of coxsackievirus A12 genome isolated from patients with different symptoms in Yunnan, Southwest China. Futur. Virol. 15, 683–691.

[ref29] Santti J. Harvala H. Kinnunen L. Hyypiä T. (2000). Molecular epidemiology and evolution of coxsackievirus A9. J. Gen. Virol. 81, 1361–1372. 10769080 10.1099/0022-1317-81-5-1361

[ref30] Song Y. Wang D. Zhang Y. Han Z. Xiao J. Lu H. (2020). Genetic diversity analysis of Coxsackievirus A8 circulating in China and Worldwide Reveals a Highly Divergent Genotype. Viruses 12 10.3390/v12101061 PMC7598191 32977444

[ref31] Sousa I. P. J. R. Oliveira M. L. A. Burlandy F. M. Machado R. S. Oliveira S. Tavares F. N. (2020). Molecular characterization and epidemiological aspects of non-polio enteroviruses isolated from acute flaccid paralysis in Brazil: a historical series (2005-2017). Emerg. Microbes Infect. 9, 2536–2546. doi: 10.1080/22221751.2020.1850181 33179584 PMC7717866

[ref32] Timmermans A. Melendrez M. C. Se Y. Chuang I. Samon N. Uthaimongkol N. (2016). Human sentinel surveillance of influenza and other respiratory viral pathogens in border areas of Western Cambodia. PLoS One 11:e0152529. doi: 10.1371/journal.pone.0152529, PMID: 27028323 PMC4814059

[ref33] Wang Z. G. Liu X. L. Yang T. T. Yi Y. (2011). Etiology of hand, foot and mouth disease in Qingdao during 2008-2009. Bing Du Xue Bao 27, 438–441. 21998955

[ref34] Wells A. I. Coyne C. B. (2019). Enteroviruses: a gut-wrenching game of entry, Detection, and Evasion. Viruses 11 10.3390/v11050460 PMC6563291 31117206

[ref35] Wu Y. Yeo A. Phoon M. C. Tan E. L. Poh C. L. Quak S. H. (2010). The largest outbreak of hand; foot and mouth disease in Singapore in 2008: the role of enterovirus 71 and coxsackievirus a strains. Int. J. Infect. Dis. 14, e1076–e1081. doi: 10.1016/j.ijid.2010.07.006 20952237

[ref36] Yang Q. Gu X. Zhang Y. Wei H. Li Q. Fan H. (2018). Persistent circulation of genotype D coxsackievirus A2 in mainland of China since 2008. PLoS One 13:e0204359. doi: 10.1371/journal.pone.0204359, PMID: 30235342 PMC6147602

[ref37] Zell R. Delwart E. Gorbalenya A. E. Hovi T. King A. M. Q. Knowles N. J. (2017). ICTV virus taxonomy profile: Picornaviridae. J. Gen. Virol. 98, 2421–2422. doi: 10.1099/jgv.0.000911, PMID: 28884666 PMC5725991

[ref38] Zhang Y. Tan X. Cui A. Mao N. Xu S. Zhu Z. (2013). Complete genome analysis of the C4 subgenotype strains of enterovirus 71: predominant recombination C4 viruses persistently circulating in China for 14 years. PLoS One 8:e56341. doi: 10.1371/journal.pone.0056341, PMID: 23441179 PMC3575343

[ref39] Zhang Y. Wang D. Yan D. Zhu S. Liu J. Wang H. (2010a). Molecular evidence of persistent epidemic and evolution of subgenotype B1 coxsackievirus A16-associated hand, foot, and mouth disease in China. J. Clin. Microbiol. 48, 619–622. doi: 10.1128/JCM.02338-09, PMID: 20018819 PMC2815627

[ref40] Zhang Y. Yan D. Zhu S. Nishimura Y. Ye X. Wang D. (2015). An insight into recombination with Enterovirus species C and nucleotide G-480 reversion from the viewpoint of Neurovirulence of vaccine-derived polioviruses. Sci. Rep. 5:17291. doi: 10.1038/srep17291, PMID: 26603565 PMC4658552

[ref41] Zhang Y. Zhu Z. Yang W. Ren J. Tan X. Wang Y. (2010b). An emerging recombinant human enterovirus 71 responsible for the 2008 outbreak of hand foot and mouth disease in Fuyang city of China. Virol. J. 7:94. doi: 10.1186/1743-422X-7-94, PMID: 20459851 PMC2885340

[ref42] Zhao H. Wang J. Chen J. Huang R. Zhang Y. Xiao J. (2022). Molecular epidemiology and evolution of Coxsackievirus A9. Viruses 14:822. doi: 10.3390/v14040822, PMID: 35458552 PMC9024771

[ref43] Zhao Y. P. Yang J. S. (2021). Epidemiological analysis of non-enterovirus 71 and non-coxsackievirus A16 enterovirus. Zhonghua Yu Fang Yi Xue Za Zhi 55, 1351–1356. doi: 10.3760/cma.j.cn112150-20210430-00431, PMID: 34749481

[ref44] Zhu B. Zhong J. Y. Xia H. M. Gong S.-T. Xiao M.-S. Xie J.-H. (2010). Etiology of hand, foot and mouth disease in Guangzhou in 2008. Zhonghua Er Ke Za Zhi 48, 127–130. 20426938

