# Supplementary material for: Genetic characterization and molecular epidemiology of Coxsackievirus A12 from mainland China during 2010–2019
Source: Front Microbiol. 2022 Dec 21;13:988538. doi: 10.3389/fmicb.2022.988538 (PMC9811122; doi:10.3389/fmicb.2022.988538)
Supplement: Supplementary file 1 [file Data_Sheet_1.docx]

Supplementary Material

# Supplementary Figures and Tables

## Supplementary Figures


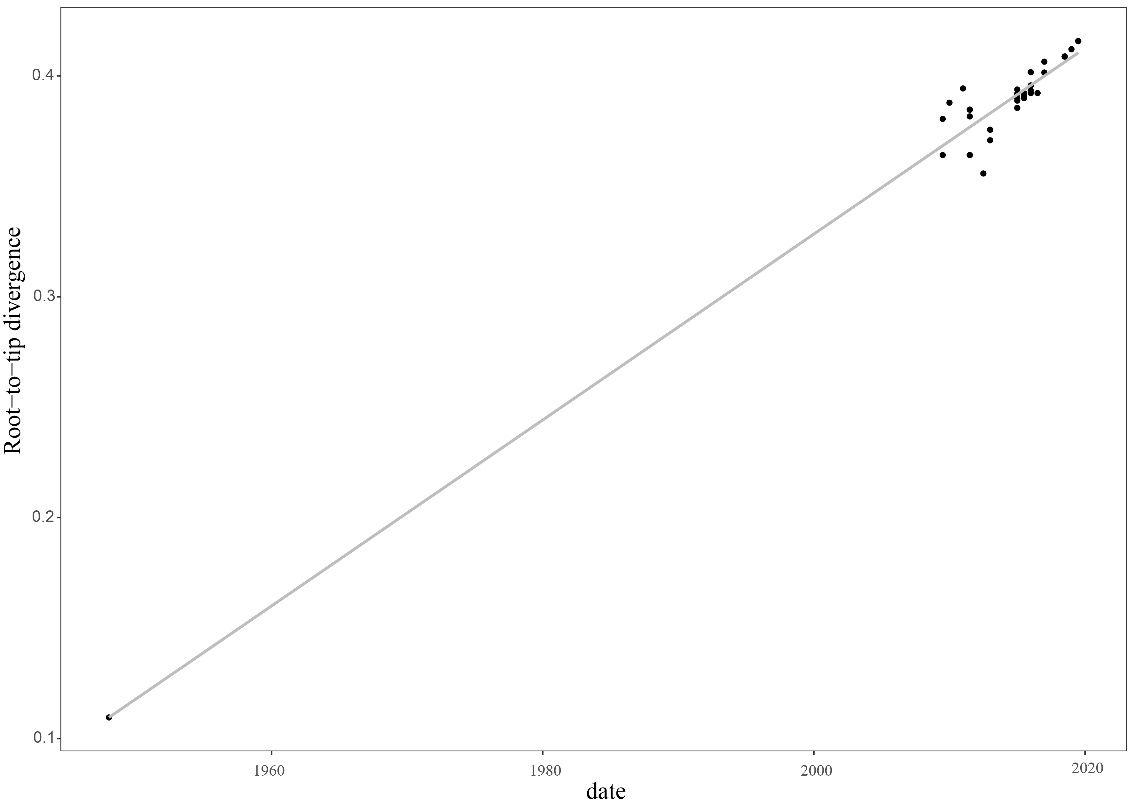


Figure S1: TempEst results for the complete 37 VP1 sequences

# 1.2 Supplementary Tables

Table S1：Clinical information of 16 CVA12 HFMD isolates from 2010 to 2019 in Mainland China

| **Lab no.** | **Gender** | **Age (years)** | **Date of sample collection** | **Sample** | **province** | **GenBank accession no.** |
| --- | --- | --- | --- | --- | --- | --- |
| CHN_CQ_2013_70 | male | 1 | 2013-03-12 | Nasopharyngeal swab | Chongqing | ON755032 |
| CHN_HAN_2017_17 | male | 2 | 2017-01-09 | Nasopharyngeal swab | Hainan | ON755031 |
| CHN_HB_2015_64 | female | 6 | 2015-07-20 | Nasopharyngeal swab | Hebei | ON755033 |
| CHN_HB_2016_16 | female | 3 | 2016-05-28 | Stool | Hebei | ON755021 |
| CHN_HN_2019_214 | female | 4 | 2019-3-18 | Stool | Henan | ON755036 |
| CHN_JX_2013_40 | male | 1 | 2013-05-02 | Nasopharyngeal swab | Jiangxi | ON755022 |
| CHN_NX_2010_83 | female | 1 | 2010-04-27 | Stool | Ningxia | ON755027 |
| CHN_SD_2015_72 | female | 1 | 2015-06-17 | Stool | Shandong | ON755035 |
| CHN_SD_2015_73 | male | 2 | 2015-06-24 | Stool | Shandong | ON755028 |
| CHN_SD_2015_81 | male | 2 | 2015-06-05 | Stool | Shandong | ON755023 |
| CHN_SD_2016_71 | male | 1 | 2016-03-19 | Stool | Shandong | ON755026 |
| CHN_SD_2016_72 | male | 3 | 2016-03-24 | Stool | Shandong | ON755030 |
| CHN_SX_2011_31 | male | 1 | 2011-05-13 | Stool | Shanxi | ON755025 |
| CHN_TJ_2015_92 | male | 1 | 2015-07-02 | Stool | Tianjin | ON755029 |
| CHN_TJ_2016_97 | female | 2 | 2016-07-11 | Stool | Tianjin | ON755034 |
| CHN_ZJ_2017_69 | male | 2 | 2017-03-16 | Nasopharyngeal swab | Zhejiang | ON755024 |

Table S2: 21 full-length VP1 sequences retrieved from GenBank

| **No.** | **GenBank number** | **Collection date** | **Region** | **Country** |
| --- | --- | --- | --- | --- |
| 1 | AF081302.1 | 1948 | / | USA |
| 2 | JX088584.1 | 2009 | Shandong | China |
| 3 | KF422142.1 | 2009 | Shandong | China |
| 4 | KF422143.1 | 2011 | Shandong | China |
| 5 | KF422144.1 | 2011 | Shandong | China |
| 6 | KF422145.1 | 2011 | Shandong | China |
| 7 | KF422146.1 | 2011 | Shandong | China |
| 8 | KF422147.1 | 2011 | Shandong | China |
| 9 | KF696708.1 | 2012 | Hunan | China |
| 10 | MH888020.1 | 2018 | Yunnan | China |
| 11 | MK061424.1 | 2018 | Yunnan | China |
| 12 | MK061425.1 | 2018 | Yunnan | China |
| 13 | MK977587.1 | 2015 | Shandong | China |
| 14 | MK977588.1 | 2015 | Shandong | China |
| 15 | MK977589.1 | 2015 | Shandong | China |
| 16 | MK977590.1 | 2015 | Shandong | China |
| 17 | MK977591.1 | 2015 | Shandong | China |
| 18 | MK977592.1 | 2015 | Shandong | China |
| 19 | MK977593.1 | 2016 | Shandong | China |
| 20 | MT495411.1 | 2019 | Yunnan | China |
| 21 | MT495412.1 | 2019 | Yunnan | China |

Table S3: The results of the Bayes test did by SpreaD3

| **From** | **To** | **Bayes_Factor** | **Posterior Probability** |
| --- | --- | --- | --- |
| SD | HUN | 1.071735748 | 0.094339623 |
| SD | YN | 0.961440612 | 0.085460599 |
| SD | CQ | 1.386507506 | 0.118756937 |
| SD | HAN | 1.270046951 | 0.109877913 |
| SD | HB | 5.396760719 | 0.344062153 |
| SD | HN | 1.043959711 | 0.092119867 |
| SD | JX | 1.401230294 | 0.119866815 |
| SD | NX | 1.113656039 | 0.097669256 |
| SD | SX | 1.043959711 | 0.092119867 |
| SD | TJ | 1.430787547 | 0.12208657 |
| SD | ZJ | 1.085674887 | 0.095449501 |
| HUN | YN | 1.155886851 | 0.10099889 |
| HUN | CQ | 1.844956617 | 0.152053274 |
| HUN | HAN | 1.5354255 | 0.129855716 |
| HUN | HB | 1.141775198 | 0.099889012 |
| HUN | HN | 1.550526507 | 0.130965594 |
| HUN | JX | 1.41599026 | 0.120976693 |
| HUN | NX | 1.401230294 | 0.119866815 |
| HUN | SX | 1.127698304 | 0.098779134 |
| HUN | TJ | 1.76606442 | 0.146503885 |
| HUN | ZJ | 4.543473659 | 0.306326304 |
| YN | CQ | 3.165742516 | 0.235294118 |
| YN | HAN | 2.204713538 | 0.176470588 |
| YN | HB | 1.844956617 | 0.152053274 |
| YN | HN | 2.054992645 | 0.166481687 |
| YN | JX | 1.342560816 | 0.115427303 |
| YN | NX | 2.480049664 | 0.194228635 |
| YN | SX | 1.797497869 | 0.14872364 |
| YN | TJ | 2.604362584 | 0.20199778 |
| YN | ZJ | 1.97335471 | 0.160932297 |
| CQ | HAN | 1.565666136 | 0.132075472 |
| CQ | HB | 1.565666136 | 0.132075472 |
| CQ | HN | 1.550526507 | 0.130965594 |
| CQ | JX | 1.198431646 | 0.104328524 |
| CQ | NX | 1.342560816 | 0.115427303 |
| CQ | SX | 0.947804123 | 0.084350721 |
| CQ | TJ | 1.327985347 | 0.114317425 |
| CQ | ZJ | 1.226971012 | 0.10654828 |
| HAN | HB | 1.876803388 | 0.15427303 |
| HAN | HN | 1.611318238 | 0.135405105 |
| HAN | JX | 1.797497869 | 0.14872364 |
| HAN | NX | 1.490352735 | 0.126526082 |
| HAN | SX | 1.016319167 | 0.089900111 |
| HAN | TJ | 1.342560816 | 0.115427303 |
| HAN | ZJ | 1.734794051 | 0.144284129 |
| HB | HN | 1.342560816 | 0.115427303 |
| HB | JX | 1.626613844 | 0.136514983 |
| HB | NX | 1.386507506 | 0.118756937 |
| HB | SX | 0.934200652 | 0.083240844 |
| HB | TJ | 1.860859133 | 0.153163152 |
| HB | ZJ | 1.641948821 | 0.137624861 |
| HN | JX | 3.030425217 | 0.227524972 |
| HN | NX | 2.6764914 | 0.206437292 |
| HN | SX | 1.099648276 | 0.096559378 |
| HN | TJ | 1.844956617 | 0.152053274 |
| HN | ZJ | 1.327985347 | 0.114317425 |
| JX | NX | 7.436165738 | 0.419533851 |
| JX | SX | 124.0604024 | 0.923418424 |
| JX | TJ | 6.878161865 | 0.400665927 |
| JX | ZJ | 7.572773282 | 0.423973363 |
| NX | SX | 1.212683625 | 0.105438402 |
| NX | TJ | 1.957156536 | 0.15982242 |
| NX | ZJ | 1.41599026 | 0.120976693 |
| SX | TJ | 2.515322351 | 0.196448391 |
| SX | ZJ | 2.005879956 | 0.163152053 |
| TJ | ZJ | 1.989595793 | 0.162042175 |
| HUN | SD | 1.550526507 | 0.130965594 |
| YN | SD | 2.154404638 | 0.173140954 |
| CQ | SD | 1.5354255 | 0.129855716 |
| HAN | SD | 2.087953008 | 0.168701443 |
| HB | SD | 8.177642645 | 0.442841287 |
| HN | SD | 1.989595793 | 0.162042175 |
| JX | SD | 4.128266096 | 0.286348502 |
| NX | SD | 1.75040893 | 0.145394007 |
| SX | SD | 2.28947186 | 0.182019978 |
| TJ | SD | 1.719219624 | 0.143174251 |
| ZJ | SD | 2.022207371 | 0.164261931 |
| YN | HUN | 2.121089865 | 0.170921199 |
| CQ | HUN | 1.445622294 | 0.123196448 |
| HAN | HUN | 1.596061852 | 0.134295228 |
| HB | HUN | 1.475404745 | 0.125416204 |
| HN | HUN | 1.657323321 | 0.138734739 |
| JX | HUN | 5.450021921 | 0.346281909 |
| NX | HUN | 1.460494646 | 0.124306326 |
| SX | HUN | 2.221573695 | 0.177580466 |
| TJ | HUN | 1.688191503 | 0.140954495 |
| ZJ | HUN | 6.147658671 | 0.374028857 |
| CQ | YN | 1.198431646 | 0.104328524 |
| HAN | YN | 1.357172907 | 0.116537181 |
| HB | YN | 1.342560816 | 0.115427303 |
| HN | YN | 1.226971012 | 0.10654828 |
| JX | YN | 182.8381185 | 0.94672586 |
| NX | YN | 1.386507506 | 0.118756937 |
| SX | YN | 1.386507506 | 0.118756937 |
| TJ | YN | 1.41599026 | 0.120976693 |
| ZJ | YN | 1.490352735 | 0.126526082 |
| HAN | CQ | 1.626613844 | 0.136514983 |
| HB | CQ | 1.626613844 | 0.136514983 |
| HN | CQ | 1.719219624 | 0.143174251 |
| JX | CQ | 3.973006858 | 0.278579356 |
| NX | CQ | 1.97335471 | 0.160932297 |
| SX | CQ | 3.107414168 | 0.231964484 |
| TJ | CQ | 1.941001101 | 0.158712542 |
| ZJ | CQ | 2.137724923 | 0.172031077 |
| HB | HAN | 1.327985347 | 0.114317425 |
| HN | HAN | 1.626613844 | 0.136514983 |
| JX | HAN | 8.552974064 | 0.453940067 |
| NX | HAN | 1.876803388 | 0.15427303 |
| SX | HAN | 2.897802716 | 0.219755827 |
| TJ | HAN | 1.97335471 | 0.160932297 |
| ZJ | HAN | 2.005879956 | 0.163152053 |
| HN | HB | 1.580844534 | 0.13318535 |
| JX | HB | 5.749547069 | 0.358490566 |
| NX | HB | 1.78176068 | 0.147613762 |
| SX | HB | 2.137724923 | 0.172031077 |
| TJ | HB | 1.580844534 | 0.13318535 |
| ZJ | HB | 1.596061852 | 0.134295228 |
| JX | HN | 7.538424366 | 0.422863485 |
| NX | HN | 2.28947186 | 0.182019978 |
| SX | HN | 2.42750352 | 0.190899001 |
| TJ | HN | 1.475404745 | 0.125416204 |
| ZJ | HN | 1.565666136 | 0.132075472 |
| NX | JX | 2.99226164 | 0.225305216 |
| SX | JX | 1.490352735 | 0.126526082 |
| TJ | JX | 1.50533876 | 0.12763596 |
| ZJ | JX | 1.475404745 | 0.125416204 |
| SX | NX | 2.306561718 | 0.183129856 |
| TJ | NX | 1.520362966 | 0.128745838 |
| ZJ | NX | 1.565666136 | 0.132075472 |
| TJ | SX | 1.565666136 | 0.132075472 |
| ZJ | SX | 0.920630079 | 0.082130966 |
| ZJ | TJ | 1.75040893 | 0.145394007 |
